# Traditional Uses of Medicinal Plants in South-Western Part of Lithuania

**DOI:** 10.3390/plants11162093

**Published:** 2022-08-11

**Authors:** Birutė Karpavičienė

**Affiliations:** Laboratory of Economic Botany, Nature Research Center, Akademijos Str. 2, 08412 Vilnius, Lithuania; birute.karpaviciene@gamtc.lt

**Keywords:** ethnobotany, folk medicine, traditional knowledge, traditional medicine

## Abstract

Changes in people’s dependence on the resources of the immediate natural environment and in the ways in which information is transmitted may result in the loss of some of the traditional knowledge of plants and their uses. In order to investigate and preserve this knowledge, a comprehensive ethnobotanical study was carried out in a little-studied ethnographic region. Knowledge about the plants used for treatment was collected through open-ended and semi-structured interviews in villages and small rural settlements in southwestern Lithuania. In total, 30 informants reported 103 plant and 1 lichen species. Although the survey was carried out in a small area, up to five local names per species were recorded. The most frequently used species were *Matricaria chamomilla*, *Tilia cordata*, *Artemisia absinthium* and *Plantago major*. The largest number of plant species was used to treat digestive and respiratory system disorders. Wild plants were mentioned in 71.0% of all use reports, while a relatively higher proportion of cultivated plants was recorded among the new uses. Decoction and infusion were the most commonly used, while some unusual preparations have been recorded in past uses. Research showed that the diversity of plant species used for healing has declined over the last 20 years and that part of traditional ethnobotanical knowledge is disappearing.

## 1. Introduction

Traditional knowledge acquired over centuries, adapted to the local culture and environment and passed on from one generation to the next must be respected and preserved, while synergies between modern science and indigenous knowledge must be encouraged [1]. Accordingly, the number of articles on ethnobotany and herbalism increased 6.3-fold at the beginning of the 21st century, and modern ethnobotanical research has recently gained more attention in Central Europe [2,3]. However, most of these studies geographically cover African and Asian countries and are still relatively rare in Northern Europe, the Baltics and other former Soviet bloc countries [2,3]. Until the 21st century, ethnobotanical research in Lithuania was dominated by the ethnologist’s view of folk medicine, where the only thing that mattered was which herb cured which disorder [4]. In the last two decades, when pharmacy students at the Lithuanian University of Health Sciences began to choose ethnopharmacological research as the basis for their Master’s thesis, research on traditional medicine in Lithuania has intensified. However, such works usually remain unpublished.

The diversity of plant species used in traditional medicine depends on the diversity of the regional flora, the availability and accessibility of plant resources and the associated knowledge of their use as herbal medicines [5]. The spontaneous Lithuanian flora includes about 1350 vascular plant species [6]. According to Naumavičius [7], a third of these species are considered phytotherapeutically effective (259), prospective (112 species) or potential (87 species). However, not all these species are common and occur throughout Lithuania.

Access to the natural environment is essential to preserve traditional practices and knowledge about the use of wild plants [8]. Although some of the population still have access to natural resources and diverse knowledge of the medical use of plants, the majority relies on a very narrow selection and a rather restricted herbal landscape [9]. Changes in the landscape or in the abundance of plant resources can lead to changes in the ethnobotanical repertoires held by people and a loss of local knowledge [10]. On the other hand, the use of herbal medicinal products and supplements increased significantly over recent decades [11]. This renewed interest in traditional herbal medicine in more developed societies can be attributed to changes in lifestyles, with a growing demand for natural products [12]. However, the transmission of knowledge through texts and other media containing local as well as non-local knowledge has a more immediate and more prolonged effect than oral transmission [13]. With the popularisation of certain complementary and alternative remedies for human health, the knowledge of some intensively marketed species has increased [14].

In order to secure the survival of knowledge on how to use locally grown plants, more attention on the regional level needs to be given to preserving and supporting the distribution of such place-specific knowledge [15]. Changes in people’s dependence on the resources of the immediate natural environment and in the ways in which information is transmitted may result in the loss of some of the traditional knowledge of plants and their uses. In order to preserve this knowledge, a comprehensive ethnobotanical study was carried out in a relatively little-studied ethnographic region. The aim of this study was (1) to document and analyse local knowledge on the current and past uses of medicinal plants and (2) to assess historical changes in medicinal plant use.

## 2. Results and Discussion

### 2.1. Diversity of Plants Used for Treatment

A total of 103 vascular plant and 1 lichen species were recorded in this study. A total of 136 vernacular plant names were registered (1.3 names per species), of which 48 (35.3%) correspond to officially approved Lithuanian genus names. This group included relatively more cultivated plant species (27.1%) and trees or shrubs (20.8%) than the group of plants named with specific vernacular names (13.6% and 6.8%, respectively). Apart from simple linguistic variations, six vernacular plant names are not recorded in any literature sources or databases. Most of the medicinal plants were registered under one vernacular name, while *Plantago major* and *Symphytum officinale* L. were registered under five names each, none of which corresponded to the officially approved Lithuanian names of these species. Vice versa, the five vernacular names referred to two or three different species each. Even 14 vernacular plant names coincided with the official Lithuanian names of another plant genus. Such mix-ups of names can be a trap for inexperienced or inattentive researchers. According to Łuczaj [16], in ethnobotanical studies, this type of error is the most common in species identification.

The species belonged to 52 families, of which Asteraceae, with 17 species, was the most frequent, followed by Rosaceae (11 species) and Lamiaceae (8 species, Table 1). These three families have the largest number of medicinal plant species in the native flora of Lithuania [7]. However, no species belonging to the Fabaceae family were recorded in this study, although it is the fourth most abundant family in Lithuania. According to Leonti [13], the plant families belonging to the Euasterids (e.g., Asteraceae, Apiaceae, Lamiaceae, Solanaceae) are generally overused, while families belonging to the Poales (e.g., Poaceae, Cyperaceae) are underrepresented in medicinal floras. Accordingly, only one species belonging to Poales, *Briza media*, was recorded in this study. Two species, namely *Cirsium vulgare* (Savi) Ten. and *Ranunculus repens* L., reported by the informants, were not identified as medicinal [7].

The most frequently mentioned species were *Matricaria chamomilla* with 34 UR, reported by 16 informants, and *Tilia cordata*, with 25 UR, reported by 19 informants, followed by *Artemisia absinthium*, *Plantago major*, *Acorus calamus*, *Vaccinium vitis-idaea*, *Thymus pulegioides* and *Vaccinium myrtillus* L. (Table 2). These plant species, with the exception of the last three, are easily accessible as they are very commonly occurring or cultivated in the immediate environment. The top eight species were used to treat the most common health problems: digestive, respiratory and urinary system disorders, wounds and infections. According to Petkevičiūtė and Mekas [4], the most popular plant species used in Lithuania in both modern and traditional medicine are *Matricaria recutita*, *Thymus vulgaris* L., *Mentha piperita* L., *Calendula officinalis* L., *Echinacea purpurea* (L.) Moench., *Valeriana officinalis* L., *Melissa officinalis* L. and *Leonurus cardiaca* L. However, most herbal remedies and teas sold in pharmacies originate from imported raw materials [17].

### 2.2. Categories of Disorders

The 487 use reports were divided into 14 groups according to the disorders they were indicated to treat. The largest proportion of UR (25.9%) was recorded, and the highest number of plant species (37) was used to treat digestive system disorders (Table 3). Despite the fact that the use of more than half of the plant species in the treatment of this group was mentioned only once, F_ic_ was the highest among all the disorder groups. This was due to the frequent reference to several species, namely *Artemisia absinthium*, *Matricaria chamomilla*, *Menyanthes trifoliata* L. and *Acorus calamus*.

For treatment of respiratory system disorders, 25 species were used, with *Tilia cordata*, *Thymus pulegioides* L. and *Tussilago farfara* L. being the most commonly mentioned species. Urinary tract disorders were the most prevalent among the genitourinary system disorders treated with medicinal herbs. These were most often treated with *Vaccinium vitis*-*idaea* and *Silene vulgaris* (Moench) Garcke. Various injuries were treated with eight plant species, the most popular being *Plantago major*. *Solanum tuberosum* was used to treat six categories of disorders, *Matricaria recutita*, *Mentha*, *Ruta graveolens* and *Thymus pulegioides* five. Among the 20 most frequently recorded plant species (according to UR), *Menyanthes trifoliata* was the only species used to treat only one group of disorders, namely digestive system disorders.

In most categories of disorders, more than half of the species were mentioned only once, with the single-mentioned items index ranging from 0.20 to 0.71 (0.49 on average, Table 3). The higher this index, the more single-mentioned species were listed in the disorder category and the higher the disagreement between informants, while low values of informant consensus factor indicate low medicinal plants homogeneity within a category of disorders. Only in four categories of disorders, namely digestive system, infections/infestations, respiratory system and injuries, the consensus factor exceed 0.6 (Table 3). This means that informants rely more on plants to treat these categories of ailments. Meanwhile, the low F_ic_ values in the other categories of disorders suggest that either the plants are chosen randomly or informants do not share information about their use [18].

### 2.3. Usual and Unusual Preparation Methods

The most commonly used for treatment were the whole aerial part of the plant (39 species, 28.3% of UR), the flowers or inflorescences (20 species, 22.8% of UR), the leaves (20 species, 16.2% of UR) and the roots or rhizomes (18 species, 16.8% of UR). Only *Plantago major* has been reported to use the whole plant with above- and below-ground parts prepared as an aqueous decoction to treat diarrhoea and macerated in alcohol for the external treatment of joint pain. Most of the plant species (60%) were prepared for internal use, 17.1% for external and 22.9% for both internal and external use. The main methods of preparation were decoction (36.5% of UR) and infusion (28.6% of UR). Only in one case, when referring to the preparation of *Matricaria chamomilla* flowers, did the informants point out a significant difference in the therapeutic effect between the decoction and the infusion. According to six informants, the decoction inhibits diarrhoea, while the infusion has the opposite effect and is laxative. The use of *M. chamomilla* as a laxative is only registered in Moroccan and Greek traditional medicine, without indicating the importance of the method of preparation [19,20].

The harder parts of the plant (roots, rhizomes, stems, bark, dried fruit) were often prepared as decoctions or macerated in alcohol. When fresh, the leaves were usually used externally to treat minor injuries or swellings. Sometimes fresh leaves were eaten with salt (*Artemisia absinthium*, *Ruta graveolens*) or honey (*Aloe arborescens* Mill.). Fresh leaves of *Tanacetum balsamita* L., *T. parthenium* (L.) Sch.Bip and *Ruta graveolens* were mixed into an omelette and used to treat diarrhoea or other digestive disorders. A Renaissance Swiss physician Konrad Gesner called *T. balsamita* ‘ovaria‘, because it leaves were used as a spice for egg dishes in the kitchen [21]. Moreover, the use of *T. balsamita* and *R. graveolens* to aromatise a specific type of omelettes as uncommon is recorded in Italy [22], while the use of *T. parthenium* for omelette preparation is prevalent among Slovenians in Northeast Italy [23]. In Slovenia, egg omelette and fried eggs are used medicinally for the treatment of gastrointestinal problems and dysmenorrhea when prepared with the following plants: *Achillea millefolium*, *Ruta graveolens*, *Tanacetum vulgare* and *Matricaria chamomilla* [24]. In Lithuania, old believers sometimes cook *Valeriana officinalis* roots with an omelette because, according to them, it is healthy and tasty [25].

Fresh *Symphytum officinale* roots, sliced and fried in butter, were also stirred into omelettes and used to treat rupture (straining) or upset stomachs (Lithuanian “trūkis”, “skrandžio pasitęsimas”). A rupture is the folk name for abdominal pain and loss of appetite caused by overwork or the lifting of too heavy a weight. This ailment was also treated with a decoction of the roots of *Inula helenium* L. or an infusion or decoction of aerial parts of *Lysimachia nummularia* L., *Polygonum aviculare* L. and *Thalictrum lucidum* L. Some herbal remedies for this disorder, i.e., an infusion of *Agrimonia eupatoria* L., fresh leaves of *Peucedanum palustre* (L.) Moench, alcoholic maceration of the roots of *Persicaria bistorta* (L.) Samp. and the freshly grated roots of *Bryonia alba*, were mixed with fats, such as butter, lard, or rendered lardon. On the other hand, the traditional use of some of these plant species (e.g., *Lysimachia nummularia*, *Thalictrum lucidum*, *Peucedanum palustre*) has completely disappeared, as all of the above-mentioned treatments for *rupture* were in the past.

The dried or fresh roots of *Plantago major* or grated roots of ‘trūkažolė’ (‘rupture herb’) are used to prepare omelettes to treat rupture [26,27]. However, the vernacular name ‘trūkažolė’ is often the name given to different plants used to treat rupture: e.g., *Symphytum officinale* (this study), *Plantago major* [27], *Pyrola* sp., *Peucedanum palustre*, *Chimaphila umbellata*, *Parnassia palustris Centaurium erythraea* and others [28]. This is a serious trap for inexperienced researchers, as ‘trūkažolė’ is the official Lithuanian name for *Cichorium inthybus*.

In the case of a rupture, there is even more confusion, as the word *trūkis* is also used to refer to a hernia. Similar confusion arises in Latvian, where the word *trūce* also means both folk disease ‘rupture’ and hernia. Sile et al. [29] even mention 22 plant species used to treat *hernia* in 19th-century Latvia. According to Sõukand and Raal [30], almost every problem related to the stomach area (except severe diarrhoea) can be called by this name.

Although, according to Pennacchio et al. [31], the worldwide use of plant-derived smoke for medicinal applications outnumbers all other uses, incense was one of the rarest of plant uses in our study. According to Petkevičius [32], incense is a rather specific treatment for fright. Similarly, in this study, *Prunella vulgaris* L. was used to incense a person scared of snakes or babies who were not sleeping well. Moreover, an infusion of the aerial part of *Prunella vulgaris* could also be prepared for the same purpose and used as a tea or bath. Similar to *Prunella vulgaris*, a decoction of the aerial part of *Thymus pulegioides* was used for bathing babies with sleep problems.

### 2.4. Selection of Medicinal Plants

Most of the recorded medicinal plants (61 species or 58.7%) were found in the close environment (gardens, meadows, pastures and fields) and could be easily accessed if needed. Although a positive correlation between the accessibility or availability and perceived usefulness of plant species has been repeatedly demonstrated [5], the use of some effective and easily accessible medicinal plants was not registered in this study. One such species was *Filipendula ulmaria* L., which is traditionally used for the supportive treatment of the common cold, and for the relief of minor articular pain [33]. There are several records of *Filipendula ulmaria* used for the treatment of sore joints [34], gastrointestinal disorders [25], stomach pain, diarrhoea, desinter, hypertension and colds [35] in other parts of Lithuania, but not in this study. The second example of the mismatch between availability and perceived usefulness is *Sambucus nigra* L., the flowers of which are traditionally used for the relief of early symptoms of common cold [36]. *Sambucus nigra*, an invasive species in Lithuania [37], is traditionally cultivated as an ornamental species and is widespread in study sites. However, none of the informants reported medicinal use of this species, while it is one of the most commonly used plants to treat the common cold in the western part of Lithuania [38].

In addition to the species already mentioned, several other common and frequent pharmacopoeial species, such as *Betula pendula* Roth, *Elymus repens* (L.) Gould, *Plantago lanceolata* L. and *Solidago virgaurea* L., were not used for treatment, while *Achillea millefolium* L., *Equisetum arvense* L. and *Viola arvensis* Murray were mentioned only once each. Four of them, namely *Betula pendula*, *Elymus repens*, *Equisetum arvense* and *Solidago virgaurea*, are effective in treating urinary tract diseases, while *Vaccinium vitis-idaea* and *Silene vulgaris* were the most commonly used to treat these diseases in the study area.

The choice of *Silene vulgaris* as well as *Briza media* for the treatment of urinary system disorders are obvious examples of the choice of plants based on the doctrine of signatures according to the shape of the organ to be treated, i.e., the bladder. Generally, both species are very rarely utilized for medicinal purposes. Similar to this study, *Silene vulgaris* is also used to treat urinary retention in Poland [39], and *Briza media* is used by old believers from Lithuania to cure inflammation of the urinary tract [25]. Other examples of the choice of plants based on the doctrine of signatures are the white flowers of *Peonia lactiflora* and *Syringa vulgaris* used to treat vaginal candidiasis and the red flowers of *Peonia* spp. are used to treat amenorrhoea.

### 2.5. Current and Past Uses of Wild and Cultivated Plants

Of the 104 species recorded, 71.2% were wild, dominated by common and abundant species (50% of wild species), while cultivated plants accounted for 26% and were dominated by popular cultivated species (66.7%). They belong to different groups: vegetables (e.g., *Solanum tuberosum* L., *Allium cepa* L., *A. sativum* L.), ornamental (e.g., *Ruta graveolens*, *Syringa vulgaris* L.), medicinal and aromatic (e.g., *Calendula officinalis*, *Mentha* spp.) and others. Rarely non-native plant parts were bought and used for treatment. Some native species were both collected in the wild and cultivated (e.g., *Matricaria chamomilla*, *Artemisia absinthium*, *Leonurus cardiaca* L.). Interviewees mentioned that *Matricaria chamomilla* used to be collected in the fields before herbicides were used, but now it is only cultivated. According to Zenderland et al. [40], the proportion of cultivated species among plants used for medicinal purposes in 18 studies varied from 7.8% to 28.2%.

The 487 notifications of use recorded in this study were grouped according to UR time: 51 plant species (256 UR) were reported as currently used, 77 (198 UR) were used in the past, and 25 (33 UR) are new uses. Only 8 species belonged to all three 3 categories, 34 to 2 and 62 to 1. More than half of the latter, namely 36 plant and 1 lichen species, have only been used in the past. Of these species, 78.4% were wild plants. Wild plants were mentioned in 71.0% of all UR, cultivated in 28.3% and exotic plants in only 0.6%. The analysis of UR in current and past use showed a fairly similar ratio, but a relatively higher proportion of cultivated plants was recorded among the new uses, i.e., 39.4% of the cultivated and 60.6% of the wild plant species were associated with UR of new uses.

**Figure 1:**
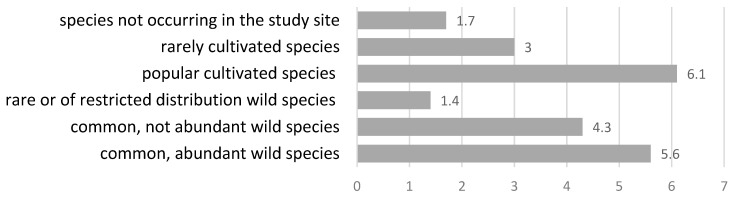
Mean use report of number per species in plant species frequency categories.

The popular cultivated and abundant wild species were used more often, with 6.1 and 5.6 UR per species, respectively, than rare wild or rare cultivated species (Figure 1). Overall, cultivated plant species were more exploited than wild ones, with an average UR per species of 5.1 and 4.7, for cultivated and wild plants, respectively, but the difference was not statistically significant (Mann–Whitney U Test, p = 0.890). Eight medicinal plant species and one lichen recorded in the survey were classified as rare or of restricted distribution species in an area. All of them were mentioned only 1–2 times, with one very clear exception of *Menyanthes trifoliata*, which was mentioned by 10 informants. This shows the importance of this species in traditional medicine in the region.

The number of species used in the past exceeded the number of currently (last two decades) used species in almost all species frequency groups. However, the average number of UR per species was higher for the currently used than for the past used (on average 5.0 and 2.6, respectively). More than half (55.3%) of the 38 species whose use was mentioned only once had been used in the past, and 28.9% were newly used species.

The true extent of knowledge loss and the exact speed at which it occurs is very difficult to assess, as different diseases are common at different ages. However, this assumption is not always correct, as informants often applied their knowledge to the treatment of their relatives at different ages. Although the last 20 years were chosen for the study, some plant species have been out of use for much longer. Despite all the exceptions, the data show that the variety of plant species used for healing is decreasing and that traditional knowledge about plants and their uses is declining.

## 3. Materials and Methods

### 3.1. Study Area

**Figure 2:**
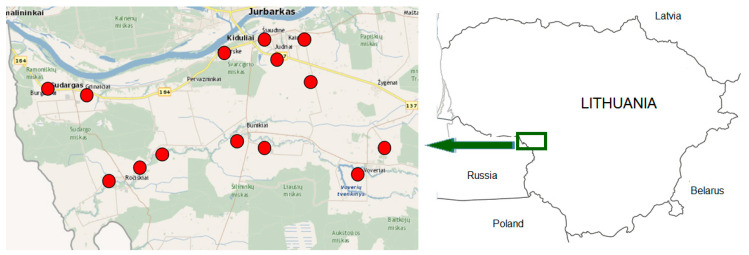
Map of the study area. Locations where data were collected are marked with a red circle.

The information was collected in villages and small rural settlements in southwestern Lithuania (Figure 2), centred on 55°2′0.2″ N and 22°43′16″ E. The territory is situated on a plain at an altitude of 10–70 m above sea level and covers 193 km^2^, of which 17% are hemi-boreal mixed broadleaved-coniferous forests. The study area is more agrarian compared to the whole territory of Lithuania, where forests account for 33.5% of the total area. The remaining part of the study area is almost entirely covered by agricultural land, with inclusions of settlements, semi-natural grasslands and small transition mires and quaking bogs. In Lithuania, as in other post-soviet Baltic states, land use has changed over the last decades, with an abundance of abandoned land, more clear-cutting of forests, while marginal areas with natural meadow vegetation are no longer mowed and are overgrown with bushes [41].

The study area is bordered to the north by the River Nemunas and to the west by the Kaliningrad Oblast, which was the northern part of East Prussia until the end of the Second World War. Since then, the use of German words has left its mark on the current everyday language of the study region [42].

In Lithuania, herbalism is seen as a female “monopoly” [43]. Although many people in rural Lithuania had at least some knowledge of herbs, it was mostly women who treated their family members, relatives and neighbours [44]. According to Gukauskiene and Juknyte [45], in Lithuania, 35% of women aged 41–70 use medicinal plants, while only 12% of men in this age group use plants for treatment.

### 3.2. Data Collection

The study was carried out in the summer of 2013. Ethnobotanical knowledge was collected through open and semi-structured interviews. Informants identified by other local people as having significant knowledge about plants and healing were interviewed. A total of 30 informants (29 women and 1 man), aged 47–91 years, were interviewed. The median age of the informants was 80 years, and only three of them were younger than 60 years. Participants who consented to take part in the study were asked to report on the use of plants: what the plants were called, what parts of the plant were collected, how they were processed and what diseases were treated. To identify changes in the use of plants, respondents were asked to indicate when a particular treatment was used: 1) all the time the same (hereinafter referred to as current use); 2) in the past (not used in the last two decades); 3) only in the last two decades (new use). If necessary, informants were asked to show fresh or dried plants or to describe the appearance and habitat of the plant as precisely as possible. If there were problems in identifying the species, informants were shown the most likely fresh plants. Some genera with more than one species occurring in the study area and difficult to identify by folk taxonomy, such as *Arctium*, *Crataegus*, *Euphrasia* and *Mentha*, were identified at the genus level. The plant names follow Plants of the World Online [46].

### 3.3. Data Analysis

The data were structured into use reports (UR), which reflect the use of a particular part of the plant, prepared or used in a particular way, for a specific medicinal category, multiplied by the number of people mentioning such use [47]. Plant species were divided into six groups according to their frequency in the study site:

(1) common and abundant wild species;
(2) common but not abundant wild species;
(3) rare or of restricted distribution wild species;
(4) popular cultivated species;
(5) rarely cultivated species;
(6) species not occurring in the study site.

The disorders mentioned by the respondents were categorised according to Cook [48], with the addition of two further categories: cancer and veterinary medicine. A single-mentioned item index (SM) for each disorder category was calculated according to Pirker et al. [49] as

SM = n_tr_/n_t_ (1)

where n_tr_ = number of taxa that are reported in each category only once, and n_t_ = number of taxa used in each category. The higher this ratio, the more single-mentioned species were listed in the disorder category and the higher the disagreement between informants.

The informant consensus factor (F_ic_) was calculated according to Heinrich et al. [50] as

F_ic_ = (n_ur_ − n_t_)/(n_ur_ − 1) (2)

where n_ur_ = number of use reports in each category, and n_t_ = number of taxa used in each category. A high value (close to 1) indicates that relatively few taxa (species) are mentioned by a large proportion of informants, while a low value indicates that the informants disagree on the taxa to be used in the treatment within a category of disorders [50].

## Figures and Tables

**Table 1:** The number of species of Lithuanian native medicinal plants in the most abundant families according to Naumavičius [7] and their number and ratio recorded in this study.

Family	Species Number		Species Ratio, %
	Naumavičius	This Study	
Asteraceae	46	12	26.1
Rosaceae	32	11	35.5
Lamiaceae	29	6	20.7
Fabaceae	26	0	0
Apiaceae	19	2	10.0
Polygonaceae	19	3	15.8
Ranunculaceae	18	2	11.1
Brassicaceae	17	1	5.9
Salicaceae	17	0	0
Ericaceae	15	5	33.3
Cyperaceae	14	0	0
Plantaginaceae	11	1	9.1
Caryophyllaceae	10	1	10.0
Orobanchaceae	10	1	10.0
Poaceae	10	1	10.0

**Table 2:** Medicinal (M) and veterinary (V) uses of wild and cultivated plants in the south-western part of Lithuania. The data were collected from interviews with 30 informants.

Latin Name	Local Name	Part Used (Use Records)	Use ^1^ (Use Records)	Preparation
*Achillea millefolium* L.	Kraujažolė	Leaf (1)	M, E: (1) wound	Fresh, crushed
*Acorus calamus* L.	Totorka	Rhizome (13)	M, I: (8) stomach problems	Decoction; maceration in alcohol with pepper or juniper ‘berries’; dried powder with water; fresh with honey
			M, E: (3) hair strengthening	Decoction
			M, I: (1) diarrhoea	Decoction
			M, I: (1) appetite stimulation	Decoction
		Leaf (1)	M, E: (1) repellent for fleas	Added in pillow and mattress stuffing
*Aesculus hippocastanum* L. *	Kaštonas, kaštanas	Flower (2)	M, E: (1) varicose veins	Maceration in alcohol
			M, E: (1) joint problems	Maceration in alcohol
		Seed (3)	M, E: (3) joint problems	Maceration in alcohol; decoction for bath
*Agrimonia eupatoria* L.	Morstytžolė	Aerial part (1)	M, I: (1) stomach problems after heavy lifting of weights	Infusion mixed with a spoon of lard or butter
*Alchemilla vulgaris* L. s.l.	Raskila	Aerial part (4)	M, I: (2) stomach problems	Infusion
			M, I: (1) diarrhoea	Decoction
			M, I: (1) inflammation, infection	Decoction
*Allium cepa* L. *	Cibulis	Bulb (5)	M, I: (2) otitis	Juice, one drop into ear
			M, I: (1) cough	Fresh with milk and honey
			M, E: (1) runny nose	Juice to lubricate the nose
			M, E: (1) hair strengthening	Grated bulb mixed with oil
*Allium sativum* L. *	Česnakas	Bulb (7)	M, I: (4) as vermifuge	Fresh
			M, E: (2) toothache	Fresh
			M, E: (1) bruises	Fresh
*Alnus glutinosa* (L.) Gaertn.	Elksnis	Bark (2)	M, V, I: (2) diarrhoea	Decoction
		Leaf (2)	M, E: (1) bruises	Fresh, young sticky
			M, E: (1) sore legs	Fresh, young sticky
*Aloe arborescens* Mill. *	Alijošius	Leaf (3)	M, I: (1) otitis	Juice into ear
			M, E: (1) wrinkles	Juice
			M, I: (1) lung infection	Fresh with honey; decoction
*Amaranthus caudatus* L. *	Burnotas	Aerial part (1)	M, I: (1) women’s health problems	Infusion
*Anethum graveolens* L. *	Krapas	Aerial part (1)	M, I: (1) blood pressure	Infusion
*Arctium* sp.	Godas, godlapis varnalėša	Root (1)	M, I: (1) stomach problems	Fresh
		Leaf (1)	M, E: (1) joint problems	Fresh
		Inflorescence (2)	M, I: (1) cough	Decoction
			M, I: (1) furuncle	Decoction
*Arctostaphylos uva-ursi* (L.) Spreng.	Meškauogė	Aerial part (2)	M, I: (2) urinary tract infections, urinary incontinence	Decoction
*Artemisia absinthium* L.	Metėlė	Aerial part, leaf (24)	M, V, I: (12) diarrhoea	Decoction; infusion
			M, I: (5) stomach problems	Infusion; fresh leaf with salt
			M, I: (4) bellyache	Decoction; infusion
			M, I: (2) appetite stimulation	Infusion
			M, I: (1) as vermifuge	Infusion
*Artemisia cina* O.Berg **	Lisninkas	Inflorescence (1)	M, I: (1) as vermifuge	Infusion
*Beta vulgaris* L. *	Cviklis, brokas	Root (2)	M, E: (2) headache	Compress with fermented juice
		Leaf (2)	M, E: (2) joint problems, sore legs	Fresh
*Bistorta officinalis* Delarbre	Vėžiukas, senšaknė	Rhizome (2)	M, I: (1) cancerM, I: (1) stomach problems after heavy lifting of weights	Maceration in alcoholMaceration in alcohol, mixed with lard or rendered lardon
*Brassica oleracea* L. *	Kopūstas	Leaf (6)	M, E: (3) bruises, swelling	Fresh
			M, E: (1) tetter	Fermented juice
			M, I: (1) stomach problems	Fresh juice
			M, E: (1) headache	Fresh
*Briza media* L.	Blakutės	Inflorescence (2)	M, I: (2) urinary tract infection	Decoction; infusion
*Bryonia alba* L. *	Perstups	Root (1)	M, I: (1) stomach problems after heavy lifting of weights	Grated fresh and fried with rendered lardon
*Bryophyllum pinnatum* (Lam.) Oken *	Paleistuvė	Leave (1)	M, I: (1) cancer	Maceration in alcohol
*Calendula officinalis* L. *	Medetka	Inflorescence (7)	M, I: (2) women’s genital tract infection	Infusion
			M, E: (2) inflammation, infection	Infusion
			M, I: (1) cough	Infusion
			M, I: (2) various problems, body cleansing	Decoction; infusion
*Callisia fragrans* (Lindl.) Woodson *	Ūsas	Leaf (1)	M, E: (1) joint problems	Maceration in alcohol
*Calluna vulgaris* (L.) Hull	Viržovė	Aerial part (1)	M, E: (1) sedative	Decoction, bath
*Cardamine pratensis* L.	Miegalė, miegžolė	Aerial part (4)	M, E: (4) insomnia	Put under pillow
*Carum carvi* L.	Kimelis	Fruit (9)	M, I: (3) diarrhoea	Decoction
			M, I: (2) induce lactation	Decoction
			M, I: (2) abdominal bloating	Decoction
			M, I: (1) intestine problems	Decoction
			M, I: (1) blood pressure	Decoction
*Centaurea cyanus* L.	Vosilka	Inflorescence (8)	M, I: (4) cough	Infusion
			M, I: (3) lung infection	Infusion
			M, I: (1) sleeping problems	Infusion
*Centaurium erythraea* Rafn	Širdažolė	Aerial part (2)	M, I: (2) heart problems, chest pain	Infusion
*Cetraria islandica* L.	Samanos	Aerial part (1)	M, I: (1) heavy cough	Infusion
*Chelidonium majus* L.	Ugniažolė	Aerial part (2)	M, I: (1) diarrhoea	Infusion
			M, I: (1) cancer	Infusion used in drops
		Latex (3)	M, E: (1) cataract	Fresh
			M, V, E: (2) warts	Fresh
*Cirsium vulgare* (Savi) Ten.	Piktdagis	Inflorescence (3)	V, I: (1) stimulate cow reproduction	Fresh inflorescence without spines
			M, I: (1) women’s health problems	Decoction
			M, I: (1) furuncles	Decoction
*Comarum palustre* L.	Vėžiažolė	Rhizome (1)	M, E: (1) leg ulcer	Maceration in alcohol
*Convallaria majalis* L.	Kanvalijos	Flower (3)	M, I: (3) heart problems	Maceration in alcohol
*Corylus avellane* L.	Riešutas	Seed (1)	M, I: (1) organism enhancing	Fresh
*Cota tinctoria* (L.) J.Gay	Arnika	Inflorescence (1)	M, I: (1) stomach problems	Decoction
*Crataegus* spp.	Gudobelė	Flower (1)	M, I: (1) heart problems	Decoction
*Daphne mezereum* L.	Žalčialunkis	Twig (1)	M, E: (1) toothache	Fresh
*Daucus carota* subsp. *sativus* (Hoffm.) Schübl. & G.Martens *	Morka	Root (1)	M, I: (1) diarrhoea	Fresh, finely grated
*Elsholtzia ciliata* (Thunb.) Hyl.	Smirdelka	Aerial part (1)	M, E: (1) joint problems	Decoction, bath
*Equisetum arvense* L.	Asiūklis, ąsuoklis	Aerial part (1)	M, I: (1) urinary tract	Decoction
*Euphorbia helioscopia* L.	Liktoriai, pienė, pienius	Latex (4)	M, E: (3) warts	Fresh
			M, E: (1) splinter	Fresh
*Euphrasia* sp.	Akišveitė	Aerial part (1)	M, E: (1) eye problems	Infusion
*Fragaria vesca* L.	Žemuogė	Aerial part (1)	M, I: (1) cough	Infusion
*Frangula alnus* Mill.	Skirpstas	Bark (1)	M, I: (1) laxative	Decoction
*Galium aparine* L.	Limpanti žolė	Aerial part (2)	V, I: (2) stimulate cow reproduction	Infusion
*Geum urbanum* L.	Žiognagė	Aerial part (1)	M, I: (1) diarrhoea	Decoction
*Glechoma hederacea* L.	Rietena	Aerial part (1)	M, I: (1) cough	Infusion
*Helichrysum arenarium* (L.) Moench	Katinpėdėlė, šiaudinė	Inflorescence (2)	M, I: (1) liver problems, jaundice	Infusion
			M, I: (1) cough	Infusion
*Hyssopus officinalis* L. *	Ysopas	Aerial part (1)	M, I: (1) abortifacient	Decoction
*Humulus lupulus* L.	Apynys	Fruit (6)	M, I: (2) insomnia	Decoction; pillow stuffing
			M, E: (2) hair strengthening	Decoction
			M, I: (2) cough	Infusion
*Hypericum perforatum* L.	Jonžolė	Aerial part (7)	M, I: (3) panacea	Decoction; infusion
			M, I: (2) bowels problems	Infusion
			M, I: (2) nerves	Infusion; maceration in alcohol
*Inula helenium* L. *	Debesils	Root (5)	M, I: (1) stomach problems after heavy lifting of weights	Decoction
			M, I: (2) bowel problems	Decoction
			M, I: (1) urinary tract problems	Decoction
			M, I: (1) body strengthening	Decoction
*Juniperus communis* L.	Kadugys	Root (1)	M, I: (1) cough	Maceration in alcohol
		Berry-like cone (1)	M, I: (1) stomach problems	Maceration in alcohol
*Knautia arvensis* (L.) Coult.	Vyriškoji moteržolė	Aerial part (1)	M, I: (1) women’s health problems	Infusion
*Lamium album* L.	Nedilginanti dilgėlė, gudmoterė	Aerial part (3)	M, I: (2) women’s health problems, vaginal candidiasis	Decoction; infusion
			M, I: (1) anaemia	Infusion
*Leonurus cardiaca* L.	Sukatžolė	Aerial part (1)	M, I: (1) sedative	Infusion
*Linum usitatissimum* L. *	Linas	Seed (11)	M, E: (5) foreign object in the eye	Linseed is placed in the eye and removed after some time
			V, I: (3) bloat in cattle	Decoction
			M, E: (1) toothache	Hot porridge, application on the cheek
			M, E: (2) furuncle, fester	Porridge in milk or water
*Lysimachia nummularia* L.	Gurgulžolė	Aerial part (2)	M, I: (2) stomach problems after heavy lifting of weights, bellyache	Infusion
*Matricaria chamomilla* L.	Remunėlė, ramunėlė, ramunaitės	Inflorescence (34)	M, I: (8) diarrhoea	Decoction
			M, I: (3) stomach problems, bellyache	Decoction
			M, E: (6) eye problems, infection	Decoction
			M, I: (6) constipation	Infusion
			M, E: (4) wound	Decoction; infusion
			M, I: (3) sore throat	Decoction; infusion
			M, I: (3) fever	Infusion
			M, I: (1) cough	Infusion
*Mentha* sp. *	Mėta, šaltmėtė	Aerial part (6)	M, I: (2) sedative	Infusion
			M, I: (1) improving digestion	Infusion
			M, I: (1) colds	Infusion
			M, I: (1) cough	Infusion
			M, I: (1) kidneys problems	Infusion
*Menyanthes trifoliata* L.	Puplaiškis	Leaf (9)	M, I: (4) stomach problems,	Decoction; infusion
			M, I: (2) diarrhoea	Decoction
			M, I: (2) appetite stimulation	Infusion
			M, I: (1) improving digestion	Infusion
		Root (1)	M, I: (1) stomach problems	Macerated in alcohol
*Paeonia* sp. *	Bijūnas	White flower (1)	M, I: (1) vaginal candidiasis	Infusion
		Red flower (1)	M, I: (1) amenorrhea	Infusion
*Petasites hybridus* (L.) G.Gaertn., B.Mey. & Scherb.	Šaukštis	Leaf (1)	M, E: (1) toe web space fungal infection	Fresh
*Peucedanum palustre* (L.) Moench	Trūkžolė	Leaf (1)	M, I: (1) stomach problems after heavy lifting of weights	Fresh with butter
		Root (1)	M, I: (1) stomach problems after heavy lifting of weights	Macerated in alcohol
*Pinus sylvestris* L.	Pušis	Bud (3)	M, I: (2) lung problems	Decoction
			M, E: (1) joint problems	Decoction, bath
*Piper nigrum* L. **	Pipiras	Seed (2)	I: (2) diarrhoea	Mixed in water
*Plantago major* L.	Traukžolė, trauklapis, raugžolė, rauglapis, rasakila	Leaf (15)	M, E: (10) wounds, bruises, swelling	Fresh
			M, I: (3) stomach problems	Decoction; infusion
			M, E: (2) felon	Fresh; salo covered with fresh leaf
		Inflorescence with seeds, seed (2)	M, I: (2) stomach problems	Decoction
		Whole plant with root (2)	M, I: (1) diarrhoea	Decoction
			M, E: (1) joint pain	Macerated in alcohol
*Polygonatum multiflorum* (L.) All.	Pakalnutė	Rhizome (3)	M, E: (3) freckles	Fresh juice
*Polygonum aviculare* L.	Takažolė	Aerial part (6)	M, I: (3) urinary tract stones	Infusion
			M, I: (2) stomach problems also after heavy lifting of weights	Decoction
			M, I: (1) bladder problems	Infusion
*Potentilla anserina* L.	Sidabražolė	Aerial part (1)	M, I: (1) strengthening potency	Decoction
*Potentilla erecta* (L.) Raeusch.	Vėžašaknis, degsnis, vėžiukas,	Rhizome (7)	M, I: (5) cancer	Maceration in alcohol
			M, I: (2) stomach problems	Decoction; maceration in alcohol
*Prunella vulgaris* L.	Juodgalvėlė	Aerial part (7)	M, E: (2) helping baby sleep peacefully	Incense of dry herb
			M, I: (2) helping baby sleep peacefully	Infusion
			M, E: (1) children‘s startle	Incense of dry herb
			M, I: (1) fright of snake	Infusion
			M, I: (1) cough	Infusion
*Quercus robur* L.	Ąžuolas	Bark (5)	M/V, I: (5) diarrhoea	Decoction
*Ranunculus repens* L.	Karpažolė	Aerial part (1)	M, E: (1) warts	Fresh, smashed
*Rheum rhabarbarum* L. *	Barbaras, rabarbaras	Petiole (1)	M, I: (1) appetite stimulation	Kompot
*Rubus idaeus* L.	Avietė	Stem, aerial part (12)	M, I: (5) fever	Decoction
			M, I: (5) cough	Decoction
			M, I: (2) sore throat, hoarseness	Decoction
*Rumex crispus* L.	Arkliarūgštė	Root (1)	M, E: (1) warts	Fresh mixed with sour cream
*Ruta graveolens* L. *	Rūta	Leaf (11)	M, I: (7) bellyache	Fresh with/without salt; fresh, cooked with omelette
			M, I: (1) hearth problems	Fresh
			M, I: (1) nerves	Infusion
			M, E: (1) hair strengthening	Decoction
			M, I: (1) abortifacient	Decoction
*Sanguisorba officinalis* L.	Kraujažolė, šakutės	Aerial part (3)	M, I: (2) diarrhoea, dysentery	Decoction
			M, I: (1) poisoning	Infusion
*Scrophularia nodosa* L.	Bervidis	Aerial part (1)	M, E: (1) radiculitis	Infusion
*Silene vulgaris* (Moench) Garcke	Pūsliukės, pūslelė	Aerial part (7)	M, I: (2) bladder inflammation	Decoction; infusion
			M, I: (1) incontinence	Infusion
			M/V, I: (2) urinary retention	Infusion
			M, I: (1) heavy menstruation	Decoction
			M, I: (1) vaginal candidiasis	Decoction
*Solanum dulcamara* L.	Virbinyčia, saldymedis	Stem (2)	M, I: (1) cough	Fresh, to chew
			M, I: (1) sore throat	Fresh, to chew
*Solanum tuberosum* L. *	Bulvė	Tuber (10)	M, I: (2) cough	Boiled unpeeled for inhalation of steam
			M, E: (2) sore throat	Boiled unpeeled, warming compress
			M, E: (2) eye inflammation	Fresh grated
			M, E: (1) bruises, burns	Fresh grated
			M, E: (2) bladder inflammation	Sit on pot with hot boiled unpeeled potatoes
			M, I: (1) stomach problems	Fresh grated, diluted
		Sprout (1)	M, E: (1) warts	Boiled, smashed
*Sorbus aucuparia* L.	Šermukšnis	Fruit (2)	M, I: (2) constipation	Fresh or dried
*Symphytum officinale* L.	Taukžolė, kaulažolė, juodšaknė, kiaulpienė, trūkžolė	Root (9)	M, E: (2) joint problems	Fresh grated and mixed with lard
			M, I: (1) bowels inflammation	Fresh or dried
			M, I: (1) low gastric acidity	Fresh or dried
			M, I: (1) stomach problems after heavy lifting of weights	Cut into slices and cooked with omelette
			M, I: (1) children’s body strengthening	Dried
			M, I: (1) nerves	Decoction
			V, I: (2) appetite stimulation	Fresh
*Syringa vulgaris* L. *	Alyva	White flower (1)	M, I: (1) vaginal candidiasis	Infusion
*Tanacetum balsamita* L. *	Moteržolė	Leaf (5)	M, I: (3) diarrhoea, nausea	Fresh, cooked with omelette
			M, I: (1) women’s problems	Infusion
			V, E: (1) to lure honeybees	Fresh, rub entrance of empty hive
*Tanacetum parthenium* (L.) Sch.Bip. *	Moteržolė	Leaf (1)	M, I: (1) bowels problems	Fresh, cooked with omelette
*Tanacetum vulgare* L.	Bitkrėslė	Inflorescence (4)	M, I: (1) cancer	Decoction
			M, I: (1) abortifacient	Infusion used for long period
			V, E: (1) insect repellent	Fresh, rub into skin or fur
			V: I: (1) diarrhoea	Decoction
*Taraxacum officinale* F.H.Wigg.	Pienė	Inflorescence (1)	M, I: (1) cough	Infusion
*Thalictrum lucidum* L.	Moteržolė, trūkžolė	Aerial part (2)	M, I: (2) stomach problems after heavy lifting of weights	Infusion
		Leaf (3)	M, I: (3) bellyache; diarrhoea	Fresh, cooked with omelette
*Thuja occidentalis* L. *	Čisai, tuja	Twigs (3)	M, E: (1) unsafe intercourse	Decoction, bath
			M, I: (1) epilepsy	Infusion
			M, I: (1) toothache	Decoction
*Thymus pulegioides* L.	Čiobrelis, čiobriukas	Aerial part (13)	M, I: (9) cough	Decoction, infusion
			M, I: (1) fever	Infusion
			M, I: (1) body strengthening	Infusion
			M, E: (1) helping baby sleep peacefully	Decoction for bath soak
			M, I: (1) heart problems	Infusion
*Tilia cordata* Mill.	Liepa	Flower (24)	M, I: (13) fever	Decoction, infusion
			M, I: (7) cough	Decoction, infusion
			M, I: (2) influenza	Infusion
			M, I: (1) bronchitis	Infusion
			M, I: (1) running nose	Infusion
		Bark (1)	M, E: (1) burning	Inner part of fresh peeled bark
*Tropaeolum majus* L. *	Žemčiūgai	Aerial part (1)	M, I: (1) cough	Infusion
*Tussilago farfara* L.	Šalpusnis	Inflorescence (10)	M, I: (7) cough	Decoction, infusion
			M, I: (1) lung infection	Infusion
			M, I: (1) asthma	Infusion
			M, I: (1) sore throat	Infusion
		Leaf (2)	M, E: (1) wounds	Fresh
			M, E: (1) sore legs	Fresh
*Urtica dioica* L.	Dilgėlė	Aerial part (5)	M, E: (5) hair strengthening	Decoction
		Root (5)	M, I: (1) lung problems, cough	Decoction
			M, E: (3) hair strengthening	Decoction
			M, I: (1) fever, contraindication high blood pressure	Decoction
*Urtica urens* L.	Mažytė dilgėlė	Aerial part (1)	M, E: (1) joint problems	Fresh, whipping
*Vaccinium myrtillus* L.	Mėlynė	Fruit, fruit with twig (13)	M, I: (7) diarrhoea	Dried fruits; decoction
			M, I: (5) eye problems	Dried fruits; decoction
			M, I: (1) trembling hands	Dried fruits; decoction
*Vaccinium oxycoccos* L.	Spanguolė	Fruit (2)	M, I: (1) appetite stimulation	Fresh
			M, I: (1) bladder problems	Fresh
*Vaccinium vitis-idaea* L.	Bruknė	Leaf (12)	M, I: (7) bladder problems	Decoction
			M, I: (2) urinary incontinence	Decoction
			M, I: (1) typhoid fever	Decoction
			M, I: (1) cough	Decoction
			M, I: (1) eye problems	Decoction
		Fruit (1)	M, I: (1) bladder problems	Fresh
*Valeriana officinalis* L.	Valerijonas, budrijolas	Root (11)	M, I: (4) nerves	Decoction
			M, I: (4) hearth problems	Decoction
			M, I: (1) sleeping problems	Decoction
			M, I: (1) stomach problems	Maceration in alcohol
			M, I: (1) headache	Decoction
*Viburnum opulus* L.	Putinas	Fruit (2)	M, I: (1) cold	Jam
			M, I: (1) abortifacient	Decoction
*Viola arvensis* Murray	Našliukės	Aerial part (1)	M, I: (1) epilepsy	Infusion

^1^ Methods of application: I = internally; E = externally. * Cultivated. ** Non-local origin.

**Table 3:** Medicinal plant species number (SN), use reports (UR), use indices and the most popular plant species according to disorders categories.

Category of Disorders	SN	UR	SM ^1^	F_ic_^2^	The Most Popular Species with UR
Digestive system	37	126	0.54	0.71	*Artemisia absinthium* 20
Respiratory system	25	70	0.56	0.65	*Tussilago farfara* 10
Genitourinary system	20	43	0.55	0.54	*Vaccinium vitis*-*idaea* 10
Skin/hair	19	39	0.58	0.53	*Urtica dioica* 8
Nervous system	16	29	0.69	0.46	*Prunella vulgaris* 5, *Valeriana officinalis* 5
Infections/infestations	14	40	0.64	0.67	*Tilia cordata* 15
Unspecified medicinal disorders	13	21	0.46	0.40	*Hypericum perforatum* 3
Musculoskeletal system	11	16	0.73	0.33	*Aesculus hippocastanum* 4
Veterinary medicine	11	20	0.45	0.47	*Artemisia absinthium* 3, *Linum usitatissimum* 3, *Quercus robur* 3
Circulatory system	10	16	0.70	0.40	*Valeriana officinalis* 4
Sensory system	8	18	0.50	0.59	*Matricaria chamomilla* 5, *Vaccinium myrtillus* 5
Injuries	9	24	0.67	0.65	*Plantago major* 10
Pain	7	9	0.71	0.25	*Allium sativum* 2, *Beta vulgaris* 2
Pregnancy/birth/puerperium	5	6	0.80	0.20	*Carum carvi* 2

^1^ SM = the single-mentioned items index. ^2^ F_ic_ = factor informant consensus index.

## Data Availability

All the data are included in the present study.
